# Revisiting the Syndecans: Master Signaling Regulators with Prognostic and Targetable Therapeutic Values in Breast Carcinoma

**DOI:** 10.3390/cancers15061794

**Published:** 2023-03-16

**Authors:** Juliana Maria Motta, Hebatallah Hassan, Sherif Abdelaziz Ibrahim

**Affiliations:** 1 Instituto de Bioquímica Médica Leopoldo de Meis, Hospital Universitário Clementino Fraga Filho, Universidade Federal do Rio de Janeiro, Rio de Janeiro 21941, Brazil; 2 Department of Zoology, Faculty of Science, Cairo University, Giza 12613, Egypt; aheba@sci.cu.edu.eg

**Keywords:** syndecans, breast cancer, tumor microenvironment, epithelial–mesenchymal transition, nuclear hormone receptors, microRNAs, exosomes, therapeutic perspectives

## Abstract

**Simple Summary:**

Syndecans (SDCs; SDC1 to 4) are integral cell surface proteoglycans that are expressed normally on various types of mammalian tissues. In cancer, SDCs’ expression is dysregulated, impacting the onset and progression of cancer by modulating pivotal signaling pathways involved in biological and molecular functions. In this review, we highlight the key roles of SDCs in breast cancer pathogenesis, addressing the prognostic value and the critical molecular regulators of SDC expression, as well as different SDC-centered therapeutic perspectives.

**Abstract:**

Syndecans (SDC1 to 4), a family of cell surface heparan sulfate proteoglycans, are frequently expressed in mammalian tissues. SDCs are aberrantly expressed either on tumor or stromal cells, influencing cancer initiation and progression through their pleiotropic role in different signaling pathways relevant to proliferation, cell-matrix adhesion, migration, invasion, metastasis, cancer stemness, and angiogenesis. In this review, we discuss the key roles of SDCs in the pathogenesis of breast cancer, the most common malignancy in females worldwide, focusing on the prognostic significance and molecular regulators of SDC expression and localization in either breast tumor tissue or its microenvironmental cells and the SDC-dependent epithelial–mesenchymal transition program. This review also highlights the molecular mechanisms underlying the roles of SDCs in regulating breast cancer cell behavior via modulation of nuclear hormone receptor signaling, microRNA expression, and exosome biogenesis and functions, as well as summarizing the potential of SDCs as promising candidate targets for therapeutic strategies against breast cancer.

## 1. Introduction

First described in 1989, syndecans (SDCs) constitute a family of transmembrane proteoglycans (PGs) expressed by diverse cells of both vertebrates and invertebrates, with heparan sulfate (HS) being the predominant glycosaminoglycan (GAG) attached to the protein core (20–45 kDa); however, chondroitin sulfate chains may also be found [1,2,3]. They display a role in the adhesion and migration capacity of cells, and they may act as co-receptors for several growth factors, cytokines, chemokines, and other signaling molecules [4,5,6].

Four members of the SDC (SDC1 to 4) family are described in mammals. SDC1 is expressed mainly on epithelial, mesenchymal cells, and some leukocytes and appears to be expressed early during embryonic development. SDC2 was initially named fibroglycan, as it was found in mesenchymal tissues, but later it was also identified in neuronal, liver, and epithelial cells. SDC3, the less studied member of this family, is expressed by neural and musculoskeletal tissues. Finally, SDC4 is the most distributed type, as it can be detected in many cell types. With the exception of erythrocytes, virtually all cells in humans express at least one SDC, and some of them express all four types [3]. Studies with SDC1 knockout mice have shown defects in epithelial cell migration. SDC2 has been reported to play an important function in angiogenesis. SDC3 knockout mice displayed neural defects and obesity resistance, while SDC4 absence led to angiogenic impairment [7].

Each SDC consists of three domains: an extracellular domain that displays low homology sequences related to the sites of carbohydrate chain binding; a transmembrane domain, which is highly conserved in distinct species; and a cytoplasmatic domain responsible for cellular signal transduction. This cytoplasmatic domain contains two conserved regions, named C1 and C2, separated by a variable (V) region, which is unique for each SDC [1]. The SDC ectodomain is constitutively released by cells via the proteolytic cleavage process (metzincin enzymes), but it may be amplified under pathological conditions, such as cancer or inflammation. In addition to promoting changes in plasma membrane dynamics, the SDC ectodomain shedding favors paracrine signaling. Another relevant aspect is that the soluble SDC ectodomain may act as a competitive molecule with membrane-bound SDCs for extracellular ligands in the microenvironment [8,9]. The remaining transmembrane and cytosolic portions of shedding SDCs are recycled. Further, the extracellular domain of SDCs possesses docking sites for integrins to form signaling complexes, which modulate the hallmarks of cancer. Integrins constitute a family of transmembrane glycoproteins formed by heterodimers of α and β subunits. They interact both with extracellular matrix (ECM) components, such as fibronectin, and with cell surface proteins [10,11]. It was shown that α5β1-integrin and SDC4 are required for cellular spreading on fibronectin [12], while αvβ3- and αvβ5-integrins with SDC1 are important for adhesion to vitronectin [13,14] and α2β1- and α6β4- integrins with distinct SDCs are essential for spreading in laminin [15,16]. Concerning breast cancer, our previous data reported that SDC1 depletion in MDA-MB-231 cells increased cell adhesion and migration on fibronectin, but the addition of exogenous IL-6 suppressed this migratory behavior. These data pointed to the fact that *SDC1* knockdown leads to an increase in β1-integrin activation status [17].

GAGs, predominantly HS, are attached to the SDC’s protein core and are able to interact with diverse growth factors and cytokines and their receptors in the tumor environment, thereby potentiating a number of signaling pathways related to tumor progression. For instance, the HS chains of SDC4 may bind to extracellular proteins, such as fibronectin, or to growth factors, such as fibroblast growth factor 2 (FGF2), or to chemokines, such as RANTES/CCL5 [18,19,20]. Therefore, they are implicated in various types of cancer development, including breast carcinomas [21]. Heparanase (encoded by *HPSE)* is an endoglucuronidase able to cleave HS chains of PGs. As a consequence, the enhanced expression and/or activation of this enzyme leads to higher shedding rates of SDCs’ GAGs within the microenvironment. There is evidence that heparanase and SDC1 work together to promote diverse signaling pathways both in tumor cells and in surrounding microenvironmental cells, such as endothelial and immune cells. For instance, hepatocyte growth factor (HGF) and vascular endothelial growth factor (VEGF) signaling are regulated by heparanase/SDC1 [22].

Breast cancer is one of the most common cancers diagnosed in women around the world, and the high mortality rate is still a challenge [23]. This cancer is primarily classified according to the histopathological characteristics (approximately 95% are ductal or lobular carcinomas) and secondarily according to the hormone receptors and other cellular markers. Based on the expression of the estrogen receptor (ER), progesterone receptor (PR), human epidermal growth factor receptor 2 (HER2), and the proliferative index Ki-67, breast cancer can be classified into different molecular subtypes: luminal A (ER-positive, PR-positive, HER2-negative, Ki-67 with less than 20%); luminal B HER2-negative (ER-positive, HER2-negative, and either PR-low or Ki-67 with more than 20%); luminal B HER-2-positive (ER-positive or PR-positive, HER2-positive or amplified, and any Ki-67); HER2-enriched (ER-negative, PR-negative, HER2-positive or amplified, and any Ki-67); and triple-negative (ER-negative, PR-negative, and HER2-negative) breast cancer [24,25,26]. It is worth mentioning that the ER has two different isoforms ERα and ERβ, which have contradictory functions on the proliferative activity of the breast [27]. Approximately 70% of breast cancer patients are ERα-positive and/or PR-negative [28]. It is noticed that ERα promotes the growth and migration of breast cancer cells, while ERβ exhibits the opposite effect [29,30]. As a result, breast tumors are classified according to ERα expression into ERα-positive or -negative breast tumors [31].

In this review, we revisit SDC expression and functions at cellular and molecular levels. Further, we discuss the potential of SDCs as attractive targets for different therapeutic strategies, given the pleiotropic roles of SDCs in breast cancer pathogenesis via regulating nuclear hormone receptor signaling pathways, microRNAs, and exosome biosynthesis.

## 2. Syndecans’ Expression in Breast Cancer Cell Lines and Its Correlation with Tumor Progression

SDCs are expressed in a variety of normal tissues, cells, and the ECM. When alterations in their expression pattern or in associated GAGs, shedding, and activation of related signaling pathways occur, SDCs influence tumorigenesis and cancer progression. In the context of breast cancer, to date, dysregulations of SDC1 and SDC4 are better understood.

Both the loss and overexpression of SDC1 have been reported during malignant progression. In general, SDC1 is upregulated in human breast cancers in comparison to normal tissue samples; therefore, it has been considered a marker of poor prognosis [32,33,34]. Moreover, in vitro studies on breast cancer cells demonstrated the role of SDC1 in promoting tumor spreading, adhesion, proliferation, and angiogenesis [14,35,36,37,38]. Sayyad and coworkers explored the brain metastasis of triple-negative breast cancer cells. They silenced SDC1 in the human MDA-MB-231 cells and overexpressed it in the murine 4T1 cells, and both cells were intracardiac injected in a mouse model. SDC1 knockdown significantly reduced tumor dissemination to the brain by modifying tumor cell secretion of cytokines and chemokines, which negatively impacts tumor cell migration across the blood–brain barrier. On the other hand, the enhanced expression of SDC1 on 4T1 tumor cells increased brain metastasis [39].

The cleavage of SDC1 in the membrane of tumor cells is also quite important for the aggressive phenotype of breast cancer cells. Nadanaka et al. in a study on the basal-like breast cancer BT-549 cell line, showed that the enzyme chondroitin 4-O-sulfotransferase-1 (C4ST-1) controls the proliferation of tumor cells via the matrix metalloproteinase (MMP)-dependent cleavage of SDC1. Moreover, they found reduced phosphorylation of S6 kinase and SUMOylated AKT in C4ST-1 knockout BT-549 cells, where cleavage of SDC1 and proliferation were suppressed [40].

Despite being considered a mesenchymal HSPG, SDC2 expression might be regulated in cancer cells. A study revealed that the inhibition of SDC2 expression in MDA-MB-231 cells diminished tumor volume and improved the survival of a xenograft mouse model [41]. Moreover, SDC2-depleted MDA-MB-231 cells display increased cell surface active β1-integrin, promoting cell spreading and adhesion, while their cell migration was reduced. This behavior is dependent on RhoGTPases [42].

Concerning SDC3, few data are conclusive on the role and relevance of this SDC in breast cancer progression. The work published by Tinholt et al. showed that endothelial, muscle, and breast cancer cells lacking SDC3 expression reduced the tissue factor pathway inhibitor I (TFPI). The TFPI is an endogenous inhibitor of TF-induced coagulation, and it has been shown to be expressed by numerous breast cancer cell lines. These findings are relevant given the importance of coagulation disorders in breast cancer patients. Additionally, this study revealed that the removal of HS chains from the SDC3 did not promote the release of TFPI in these cells, suggesting that SDC3-TFPI binding is not dependent on HS attached to SDC3 [43,44]. In another work, Wu and collaborators failed to show a correlation between SDC3 expression in breast carcinoma and lymph node metastasis [45].

Different studies show that a higher expression of SDC4 in the subgroup of ER/PR-negative and triple-negative breast cancer patients is correlated with poor survival rates. Its expression seems to be enriched in focal adhesions. Studies on breast cancer cell lines demonstrated an overexpression of SDC4 in MDA-MB-231 triple-negative breast cancer (ER-negative, PR-negative, and HER2-negative) and HER2-overexpressing SKBR3 (ER-negative, PR-negative, HER2-positive) cells, but a downexpression in less aggressive MCF-7 (ER-positive, PR-positive, HER2-negative) cancer cells [46,47]. Mundhenke and co-workers showed, in a study on MCF-7 cells, that both SDC1 and SDC4 contribute to the FGF2 receptor complex formation [48]. Corroborating these findings in breast cancer tissues, SDC1 and SDC4 expression levels are correlated with FGF receptor complex expression [49]. Furthermore, SDC4 ectodomain shedding is also relevant for breast cancer growth since it promotes cytokinesis dependent on phosphorylation in MCF-7 cells. SDC4 phosphorylation especially occurs at the G2/M cell cycle phase [46].

## 3. Tumor-Associated Stromal Cell Expression of Syndecans

In addition to the relevance of cancer cell expression of SDCs, tumor microenvironmental cells may express these PGs, thereby influencing tumor progression. Tumor-associated stromal cells have been associated not only with immunosuppression but also with tumor growth and chemoresistance in breast cancer patients [50]. The stroma is composed of various cell types, including mesenchymal stromal cells (MSCs), cancer-associated fibroblasts (CAFs), immune cells (from both lymphoid and myeloid lineages), the endothelium, and their surrounding ECM [51]. The tumor microenvironment is nowadays recognized as a crucial factor for tumor development and progression. For instance, stromal cells expressing fibroblast activation protein alpha (FAPα), fibroblast surface protein (FSP), α smooth muscle actin (αSMA), and lacking CD45 and CD11b expression that are present in the breast and lung tumor microenvironment promote tumorigenesis and create an immunosuppressive niche [52,53].

The role of CAFs has been extensively studied because these cells are a key group within the tumor microenvironment and are major producers of most ECM components [54,55]. Maeda et al. showed that the aggressive breast carcinoma MDA-MB-231 cells were capable of inducing SDC1 expression on embryonic fibroblasts in a coculture experiment. This occurrence was not seen with the less aggressive T47D cells. However, when they cultured T47D cells in contact with SDC1-transfected fibroblasts, the tumor growth was significantly increased. Additionally, fibroblasts expressing a mutant SDC1 (without HS chains) are not able to stimulate tumor growth [56]. Thus, the HS chains attached to the SDC1 are essential for stroma-induced breast cancer development.

Stromal SDC1’s role in primary tumor evolution to metastatic dissemination has also been suggested in humans. In infiltrating human breast carcinomas, SDC1 accumulation within the tumor stroma was sought to contribute to neovascularization and stromal proliferation since HS chains usually interact with diverse heparin-binding growth factors, such as FGF-2. Comparing infiltrating and local breast carcinomas, it was demonstrated that SDC1 is present in both the connective tissue and stroma of metastatic carcinomas, while absent in the stroma of normal tissue or local carcinomas [57].

In the context of the tumor microenvironment, SDC2 has been shown to be expressed on stromal cells within breast cancer tissue and positively modulates the transforming growth factor-β (TGF-β) signaling mainly through *SMAD7*, *PAI-1*, and *CXCR4* [58]. These signaling pathways are directly related to the epithelial–mesenchymal transition (EMT) and metastasis.

The relationship between SDC3 expression in the tumor microenvironment and hypoxia has been recently explored. Pietro-Fernández et al. reported an increased expression of SDC3 by tumor cells in vitro when hypoxia-inducing factor-1 (HIF-1α) is expressed after exposure to 1% oxygen. Notwithstanding, the same profile of HIF-1α inducing SDC3 expression was shown in macrophages and endothelial cells within the melanoma microenvironment [59]. Despite not being demonstrated in breast cancer environments, these data point to an important factor related to the immune cell expression of SDCs and solid tumor progression, as hypoxia is a common cancer-related event.

The expression of SDC4 is also crucial in inflammatory cells within the tumor microenvironment. Given the fact that macrophages are probably the most relevant immune cells within the tumor microenvironment, their secreted cytokines and other soluble products are of paramount importance. Wang and collaborators demonstrated that SDC4 is a coreceptor for tumor necrosis factor-α (TNF-α). Moreover, TGF-β can inhibit TNF-α-induced MMP-3 by neutralizing SDC4 and nuclear factor-κB (NF-κB) signaling. In macrophages, TNF-α stimulates SDC4 expression [60,61].

## 4. Relationship between Syndecans and Epithelial–Mesenchymal Transition

The EMT is a physiological phenomenon commonly used by tumor cells for metastatic dissemination. This biological process is a switch in which epithelial cells that are normally in contact with the basal membrane and in close contact with their neighbor epithelial cells acquire phenotypic changes to assume mesenchymal features [62]. Among this new mesenchymal phenotype, cells lose contact with their neighborhood, enhance their migratory capacity and invasiveness, their resistance to apoptosis, reorganize their cytoskeleton and polarity, and express distinct transcription factors such as *SNAIL 1/2* and *ZEB 1/2* and molecules such as N-cadherin, fibronectin, and vimentin [63,64]. Similarly, the reversal process, named the mesenchymal–epithelial transition (MET), is also essential for metastasis success. Once they reach the metastatic site, cells undergo MET to colonize and proliferate in order to form a secondary tumor [65]. Once they acquire the epithelial phenotype again, cells preferentially express E-cadherin instead of N-cadherin and produce laminin-1 and collagen IV [66].

In a study with ductal breast carcinoma in situ, D’Arcy and coworkers depleted SDC1 in MCF10A cells and observed an upregulation of EMT marker gene expression, including E-cadherin (*CDH1*), fibronectin-1 (*FN1*), and claudin-1 (*CLDN1*). Moreover, they detected an upregulation of *MMP3*, *MMP9*, and *HPSE* and a downregulation of *MMP2*, vimentin (*VIM*), and Rho associated coiled-coil containing protein kinase 2 (*ROCK2*) [67].

The EMT has been associated with cancer stemness. In breast cancer, stem cells are characterized by the expression of membrane molecules CD24 and CD44 as well as by the expression of EMT proteins and transcription factors, for instance, neurogenic locus notch homolog protein 1 (NOTCH1) and octamer-binding transcription factor 4 (OCT4) [68]. SDC1 is capable of modulating breast cancer stem cell phenotypes via interleukin (IL)-6/signal transducers and activators of transcription 3 (STAT3), Notch, and epidermal growth factor receptor (EGFR) signaling pathways [69]. IL-6 is a known mediator of the EMT process in breast carcinomas, and it was shown that SDC1 silencing suppresses IL-6 signaling pathways, resulting in less proliferative and metastatic behavior [70]. Furthermore, SDC1 and E-cadherin seem to be coordinately expressed in several cancer types, therefore affecting cancer EMT and progression [71].

Despite a lack of information regarding the association of SDC2 and the EMT in breast cancer, SDC2 emerges as a promising candidate with a relevant function in this context since its expression seems to be higher in mesenchymal than epithelial tissues [72].

Although not conducted in breast cancer cell models (the main focus of this review), the following studies presented relevant data that should be considered for new breast cancer research. In 2015, it was described that shed SDC1 is translocated to the nucleus and alters histone acetylation in bone marrow stromal cells. Moreover, GAG chains are required for nuclear translocation since the addition of exogenous heparin or the cleavage of HS chains by using heparinase III or sodium chlorate blocked its translocation to the nucleus [73]. Using fibrosarcoma cells, a study by Szatmári and co-workers showed that tumor cells also internalize SDC1, and their proliferation is prevented by the nuclear translocation of this PG. It implicates tumor cell cycle arrest with an accumulation of cells in the G0/G1 phase [74]. Later, in 2021, the same research group associated TGF-β1 stimulation with a switch of E-cadherin to N-cadherin and a loss of SDC1 in the nuclear compartment of human lung cancer cells. Additionally, it was found with B6FS fibrosarcoma cells that nuclear translocation of SDC1 suppresses the mesenchymal phenotype and invasive behavior of these cells [75].

## 5. Interplay between Syndecans and Nuclear Hormone Receptors in Breast Cancer Progression

### 5.1. Nuclear Hormone Receptors

The nuclear hormone receptor superfamily comprises 48 human nuclear receptors, which act as nodal regulators of almost all biological processes, such as metabolism, growth, reproduction, and immune and stress responses [76,77]. Based on the presence of prominent naturally occurring ligands, nuclear hormone receptors can be classified into endocrine receptors (steroid and heterodimeric receptors), adopted orphan receptors (lipid sensor and enigmatic orphans), and true orphan receptors [77,78]. They exert their regulatory functions by exhibiting direct access to DNA for target gene transcription upon ligand binding. Nuclear receptors have also emerged as pivotal players in tumor promotion or suppression in various types of cancer [79]. Thus, they were intensely studied as potential therapeutic targets in cancer, with more focus on targeting steroid nuclear receptors in hormone-dependent cancers [80]. Particularly, the ER and PR in breast cancer and androgen receptor (AR) in prostate cancer. ER and PR expression are fundamental factors in breast cancer classification and treatment strategy decisions. Notably, they are also considered critical prognostic factors in breast carcinoma [80,81]. Growing evidence supports the existence of a potent interplay between SDCs and various types of nuclear receptors, specifically ER, PR, and AR, that contribute to breast cancer progression or regression, and that crosstalk will be discussed in the next section.

### 5.2. Crosstalk between Syndecans and Steroid Nuclear Receptors in Breast Cancer

In normal mammary glands, the steroid hormones and their cognate receptors (such as ER and PR) are implicated in the growth and development of the mammary gland during puberty, as well as in breast tumor initiation and progression [82,83,84,85]. As we have mentioned earlier, accumulating clinical evidence has revealed that SDC1 overexpression is strongly correlated with ER and PR negativity, high-grade and large-size breast cancer tumors, and subsequent poor clinical outcomes [32,34,86,87,88]. Additionally, cellular localization of SDC1, either membrane-bound or cytoplasmic, in ductal carcinoma tissues in situ is related to ER-/PR-positive or -negative patients, respectively. The same study suggested that the membranous localization of SDC1 retards tumor growth while its cytoplasmic expression supports tumor progression [86]. On the other hand, regarding the involvement of another member of the SDC family as a biomarker in breast cancer, there is a conflict about the implication of SDC4 in breast cancer progression and its relation to ER/PR status. One study revealed an association of SDC4 with ER negativity [87], while another study reported a positive relation between SDC4 expression and ER/PR expression and suggested that its expression is lacking in high-grade tumors [88]. A recent study used Kaplan–Meier plotter analysis for 5000 breast cancer patients to further evaluate the prognostic value of SDC4. It was found that SDC4 overexpression is a poor prognostic factor in patients with ER-negative and ER-/PR-negative breast cancer, whereas it is a good prognostic factor for all breast cancer patients analyzed, irrespective of the molecular subtypes. Thus, the authors suggested that the prognostic value of SDC4 is breast cancer subtype-dependent [89]. Taken together, these findings highlight the prognostic significance of SDC1 and SDC4 in breast cancer.

**Figure 1:**
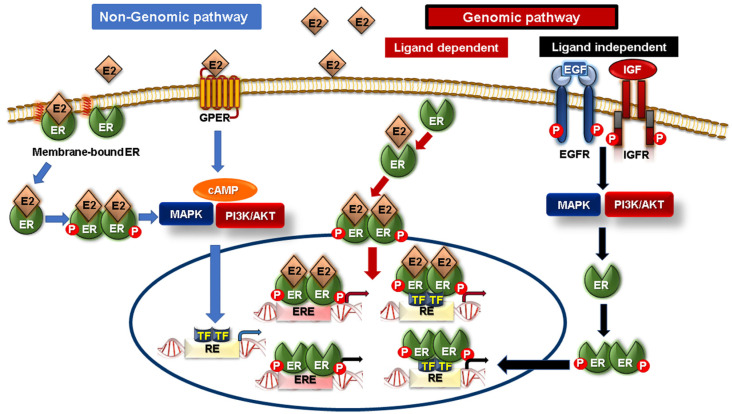
Estrogen receptor (ER) signaling pathway. Two signaling pathways are known to mediate ER signaling, including genomic (ligand-dependent and -independent) and non-genomic pathways. In ligand-dependent genomic pathways, estradiol (E2) can bind to ER to induce transcription of estrogen response element (ERE) genes. In ligand-independent genomic pathways, phosphorylation of ER is mediated by EGF/EGFR or IGF/IGFR-induced PI3K/AKT and MAPK activation. On the other hand, the non-genomic pathway is regulated by membrane-bound ER or G-protein coupled ER (GPER), whereby binding to E2 leads to activation of PI3K/AKT and MAPK signaling.

It is documented that the ER is not only nuclear but also cytoplasmic- and membranous-localized [90,91]. Generally, as depicted in Figure 1, ER signaling can be mediated through two pathways: a genomic pathway and a nongenomic pathway acting either independently or in concert [92]. In the genomic pathway, ER signaling is either ligand-dependent or -independent. In ligand-dependent ER signaling, the ER binds to its ligand estradiol (E2), promoting its dimerization and nuclear translocation. Subsequently, the E2-ER complex induces the transcription of target genes either via direct transcriptional signaling, where the ER acts as a transcription factor for target genes that have an estrogen response element (ERE), or via indirect transcriptional signaling, where the ER triggers the transcription of genes without an ERE through activating other transcription factors [93,94]. In the case of ligand-independent signaling, the ER is activated by other extracellular signaling factors, such as IGF and EGF, via their binding to their cognate EGFR and IGFR, respectively, to induce protein kinases (PI3K/AKT and MAPK pathways) and activate the ER by phosphorylation [92]. The non-genomic pathway of ER signaling, also known as the “membrane-initiated pathway”, is mediated through membrane receptors, mainly membrane-bound ER, along with G-protein-coupled ER (GPER), which can also bind to E2 [94,95]. Since the ER has no transmembrane domain as GPER, the tethering of the ER to the plasma membrane can be facilitated through their palmitoylation or myristoylation. Upon activation via binding to E2, the ER dissociates from the membrane to induce downstream signaling pathways such as PI3K and MAPK signaling pathways that rapidly regulate various critical cellular functions [93,95].

**Figure 2:**
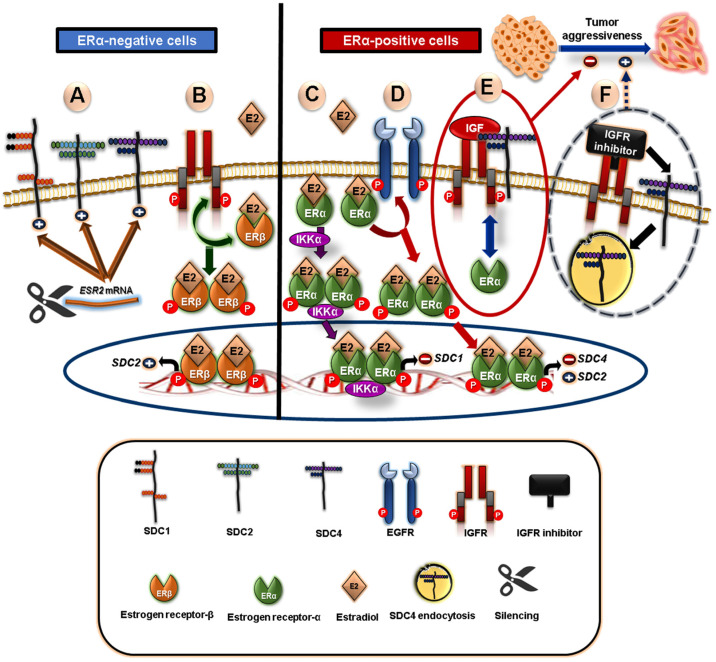
Schematic diagram showing the possible crosstalk between syndecans (SDCs) and estrogen receptors (ER) in breast cancer cells. In ERα-negative cells, (**A**) silencing of *ESR2* in MDA-MB-231 breast cancer cells induces the expression of *SDC1*, *2*, and *4*, and (**B**) estradiol (E2)-induced SDC2 expression depends on IGFR signaling crosstalk. In ERα-positive cells, (**C**) ERα activation by E2 in IKK-dependent mechanisms enhances ERα stability and transcriptional activity, leading to *SDC1* repression, (**D**) EGFR mediates E2 inhibitory action on *SDC4* expression and E2-induced *SDC2* expression, (**E**) IGFR inhibits aggressiveness of ERα-dependent MCF-7 by sustaining the expression of *SDC4*, and (**F**) IGFR colocalizes with SDC4, and that IGFR blocking results in SDC4 endocytosis and aggressiveness phenotype.

Moreover, several studies have reported that E2-ER signaling has a modulatory impact on SDC expression not only in ERα-positive but also in ERα-negative breast cancer cells (Figure 2) [96]. E2-ER signaling induces SDC2 expression while repressing SDC4 expression in both ERα-positive and -negative breast cancer cells. Mechanistically, the E2 effect on SDC expression is mediated through the interplay between ERs, EGFR, and/or IGFR. In ERα-positive cells, EGFR is a critical mediator of the E2 inhibitory impact on SDC4 expression. However, EGFR has an opposing influence on E2-induced SDC2 expression. In ERβ-positive cells, IGFR has a principal role in E2-induced SDC2 expression [91]. Moreover, IGFR is a key regulator of cell adhesion and invasion via the modulation of SDC4 and integrin expression at the protein level based on ER status, which is also strongly influenced by IGFR activation. This study suggested that IGFR plays a protective role against breast cancer aggressiveness by sustaining the expression of SDC4 and integrins for binding to fibronectin and laminin in ERα-expressing MCF-7 cells [91,97]. Notably, it has been found that IGFR colocalizes with SDC4, and blocking IGFR results in SDC4 endocytosis and loss at the cell surface. This explains the cooperation between IGFR and SDC4 in ERα-positive breast cancer cells [97]. Furthermore, SDC1 expression is inversely associated with ERα expression in ERα-positive MCF-7 cells, where the silencing of ERα induces SDC1 expression. On the contrary, ERα activation by E2 via an IKK-dependent mechanism mediates SDC1 repression [98]. Mechanistically, IKKα regulates estrogen-induced phosphorylation of ERα on Ser188, which subsequently enhances ERα stability and transcriptional activity, leading to the synthesis of repressors that mediate SDC1 downregulation [98,99,100]. Interestingly, the silencing of ERβ in MDA-MB-231 breast cancer cells induces the expression of *SDC1*, *2*, and *4* [101].

The AR is another relevant steroid receptor expressed in breast cancer [102]. Being overexpressed in primary and metastatic mammary cancer, the AR emerges as a new biomarker and a promising therapeutic target in breast cancer [103]. The transcriptional activity of the AR is modulated by androgen (such as testosterone and dihydrotestosterone) engagement [104]. SDC1 and the AR have been identified as molecular markers with a high expression in breast cancer tissues compared to normal tissues [34]. Previous studies revealed crosstalk between the AR and SDCs, where testosterone stimulation of murine mammary carcinoma cells hinders SDC ectodomain expression associated with EMT, a switch from heparin-dependent to integrin-dependent fibronectin adhesion, and enhanced cancer cell proliferation [105,106,107]. Interestingly, AR and stromal SDC1 expression are altered concomitantly by subjecting breast cancer to agonists for the ER, PR, and/or AR in an animal model [108].

### 5.3. Crosstalk of Syndecans with PPAR-Gamma “Adopted Orphan Nuclear Receptors”

Adopted orphan nuclear receptors were initially recognized due to their structural similarity to the endocrine receptors with no identified endogenous ligands. Thus, they are referred to as orphan receptors. However, they become “de-orphanized” after their ligands’ discovery and become known as “Adopted orphan nuclear receptors” [78]. One member of the adopted orphan nuclear receptor group that has recently been extensively studied in breast cancer is the “Peroxisome proliferator-activated receptor (PPAR)”. It possesses three isoforms: PPAR-α, PPARβ/delta, and PPAR-gamma [109]. They are structurally homologous to ERα and functionally transcribe some target genes of ERα [77]. PPAR-gamma in particular is the most widely studied isoform regarding its anti-proliferative, anti-invasion, anti-migratory, and pro-apoptotic effects in breast cancer cells [110,111,112,113,114]. Furthermore, its high expression is considered a good prognostic indicator of breast carcinoma [115]. PPAR-gamma is also widely known as the master regulator of adipogenesis and adipokine synthesis [116] and can be activated via natural (including long-chain polyunsaturated fatty acids and prostanoids) or synthetic ligands (thiazolidinediones (TZDs), also called glitazones) [117,118]. In MCF-7 cells, the transcriptional level of SDC1 is upregulated upon stimulation of PPAR-gamma with natural (n-3 polyunsaturated fatty acids; n-3 LDL or DHA) or synthetic (troglitazone) ligands, resulting in apoptosis. SDC1, as a target gene for PPAR-gamma transcriptional activity, was verified by a luciferase reporter assay [113]. Mechanistically, SDC1 overexpression triggers cell death by blocking the MEK/Erk/Bad signaling pathway in vitro in MCF-7 and SK-BR-3 cells and in vivo models [119]. Another study showed that SDC1 acts as a receptor for very low-density lipoprotein (VLDL) uptake, inducing adipocyte differentiation via PPAR-gamma activation in intradermal adipose tissue [120]. Since adipose tissue plays a pivotal role in breast cancer progression [121], the interplay between SDC1 and PPAR-gamma needs further investigation in order to establish new potential therapeutic strategies for breast cancer. Further, determining whether the expression of other SDC family members could be regulated by PPAR-gamma signaling is required for further studies in breast cancer.

## 6. MicroRNA-Dependent Syndecan Regulation and Breast Cancer

MicroRNAs (miRNAs) are a class of small noncoding RNAs (~19–24 nucleotides) that are involved primarily in gene expression repression via specific base-pairing with their target gene’s 3′ untranslated region (UTR) [122]. The degree of seed sequence (6–7-nucleotide) complementarity in the 5′ end of the single-stranded miRNAs either leads to the degradation of their mRNA targets or the repression of the translational process [123,124,125]. However, miRNAs can also act as positive regulators of gene expression via binding to the 5′ UTR of their target genes [124,126,127]. miRNAs can be classified into oncogenes or tumor suppressors according to their target genes [125,128,129,130]. miRNA sequences from 271 organisms revealed 38 589 hairpin precursors and 48 860 mature miRNAs according to the latest miRBase release (v22) [131]. Several studies underscored the clinical significance of miRNAs serving as diagnostic, prognostic, and predictive markers for a wide range of tumor entities [132,133]. Mechanistically, dysregulated miRNA expression via regulation of their targets can affect hallmarks of cancer, namely cell proliferation, apoptosis, adhesion, migration, invasion, and metastasis, as is evident from both in vitro and in vivo studies [129,134,135,136,137,138].

The regulation of the four SDC family members’ expression can be affected by the aberrant expression of miRNAs in different diseases, including breast cancer, consequently affecting cancer cell signaling and behavior [125]. To the best of our knowledge, miRNA-dependent *SDC1*, *2*, and *4* expression regulation has been reported in breast cancer. However, no study has yet revealed this miRNA-dependent regulation for *SDC3*, which has been shown to be expressed in mammary carcinoma tissues [45] and strongly linked to the overall survival of breast cancer patients [139].

**Figure 3:**
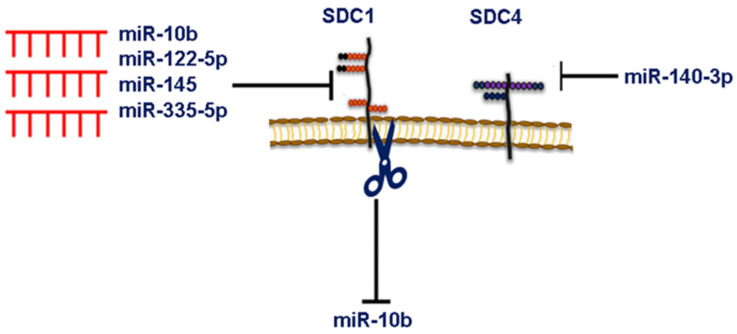
Bidirectional relations between SDCs (*SDC1* and *4*) and miRNAs in breast cancer. *SDC1* expression can be directly or indirectly targeted by miR-10b, miR-122-5p, miR-145, and miR-335-5p, whereas *SDC4* is directly targeted by miR-140-3p. On the other hand, downregulation of *SDC1* results in reduced miR-10b expression. The interplay between SDCs and miRNAs ultimately affects breast cancer cell behavior (e.g., cell proliferation, adhesion, migration, and invasion).

Data from our group revealed that the silencing of *SDC1*—identified as a direct downstream target of miR-10b, evidenced by a luciferase reporter assay and qPCR—promotes breast cancer cell migration and invasiveness through Rho-GTPase- and E-cadherin-dependent mechanisms [134]. These findings were further corroborated in another study reporting that the silencing of ERβ in MDA-MB-231 cells results in significantly altered expressions of miR-10b and miR-145, associated with changes in cell behavior and ECM composition, including *SDC1* expression [129]. Interestingly, a recent study reported that SDC1 can act as an upstream regulator of miR-10b expression, where *SDC1* silencing led to the downregulation of miR-10b in both MCF-7 and MDA-MB-231 cells [140]. This may suggest that a mutual regulation exists between the expression of both *SDC1* and miR-10b in breast cancer (Figure 3). miR-122-5p overexpression or liver-cell-derived exosomal miR-122-5p enhances the human breast cancer MCF-7 cell motility via directly targeting the *SDC1* 3′ UTR [141]. An overexpression of miR-335-5p retards the growth, invasion, and migration of breast cancer cells via the direct targeting of *SDC1* (Figure 3) [142]. The metastasis suppressor Raf-1 kinase inhibitory protein (RKIP/PEBP1) downregulated *SDC2* mediated by the suppression of the high mobility group AT-hook 2 (HMGA2) in a miR-200b-independent manner, leading to blunted breast tumor growth and metastasis and enhanced apoptosis in a mouse xenograft model of breast cancer [41]. The SDC binding protein (SDCBP), also known as ‘‘syntenin” was originally identified as an interacting molecule with the cytoplasmic domain of SDC through its PDZ-containing domains, which mediated cytoskeletal signaling [143]. An overexpression of miR-135b-5p retards breast cancer cell growth, the EMT, migration, invasion, and metastasis via the direct targeting of *SDCBP* in vitro and in breast tumor xenografts [144]. The same study provides clinical evidence that the miR-135b-5p/SDCBP axis plays a crucial role in the metastasis of early-stage breast cancer [144]. Only one recent study by Dr. Götte and his group shows that *SDC4* is a target of miRNA in breast cancer (Figure 3). They demonstrated that *SDC4* is a direct target of miR-140-3p, whose overexpression phenocopies the antitumor effects of *SDC4* silencing, leading to reduced adhesion, migration, and invasiveness of mammary carcinoma MDA-MB-231, SKBR3, and MCF-7 cells via modulation of *FN*, *FAK*, *MMP2*, and *HPSE* expression [47]. Future studies are necessary to delineate the functional interplay between the expression of SDC family members and new miRNAs, as well as the signaling events altered, thereby influencing the hallmarks of breast cancer.

## 7. Syndecans Regulate Exosome Biogenesis and Composition

Exosomes are nanosized (30–150 nm) membrane-derived extracellular vesicles (EVs) of endosomal origin encompassing signaling biomolecules, including lipids, mRNA, miRNA, long non-coding RNAs, and proteins [145,146]. These vesicles not only act as potent mediators of local intercellular communication within the tumor microenvironment, but they are also able to circulate in the blood and deliver their cargo content to prepare the premetastatic niches at distant sites [145]. Interestingly, it has been discovered that the pre-metastatic niche is prepared via cancer-derived exosome uptake by organ-specific cells, a process called “organotropic metastasis” [147]. A number of studies have shed light on exosomes as circulating drug delivery vehicles and diagnostic markers, thus emerging as novel theranostic tools for cancer as well as prognostic and predictive disease markers [146,148]. It has become apparent that exosome content and biogenesis mechanisms are varied and are cell-context dependent. Exosomes are biosynthesized in three main steps involving plasma membrane invagination (to form primary endocytic vesicles and early endosomes), the generation of multivesicular bodies (MVBs) containing intraluminal vesicles (ILVs; via maturation of early endosomes into late endosomes), and secretion (upon fusion of MVBs with plasma membrane) [149]. It has been elegantly reported that, in MCF7 cells, SDC, through syntenin with ALIX (the endosomal sorting complex required for transport (ESCRT)-interacting protein), acts as an important regulator of membrane trafficking and exosome biogenesis [150,151]. Syntenin interacts via its PDZ domain with the intracellular domain of SDCs or via its N-terminal domain with ALIX [150]. The interaction of syntenin with SDCs in endosomes either facilitates cell surface recycling of SDCs (in a small GTPase ADP ribosylation factor 6 (ARF6)/phosphatidylinositol (4)-phosphate 5-kinase-dependent mechanism mediated by the direct interaction of syntenin with PIP2) [152] or directs SDC1 into ILVs within MVBs, which fuse with the plasma membrane to secrete exosomes [150]. The latter process is also regulated by ARF6 activation and its effector lipid-modifying enzyme phospholipase D2 [151,153]. Importantly, after SDC HS chains are trimmed by heparanase enzymatic activity and their extracellular domains are prone to proteolytic cleavage, a C-terminal fragment of SDCs is generated to induce endosomal budding and exosome secretion [154].

## 8. Syndecans as Targets for Breast Cancer Therapies

**Figure 4:**
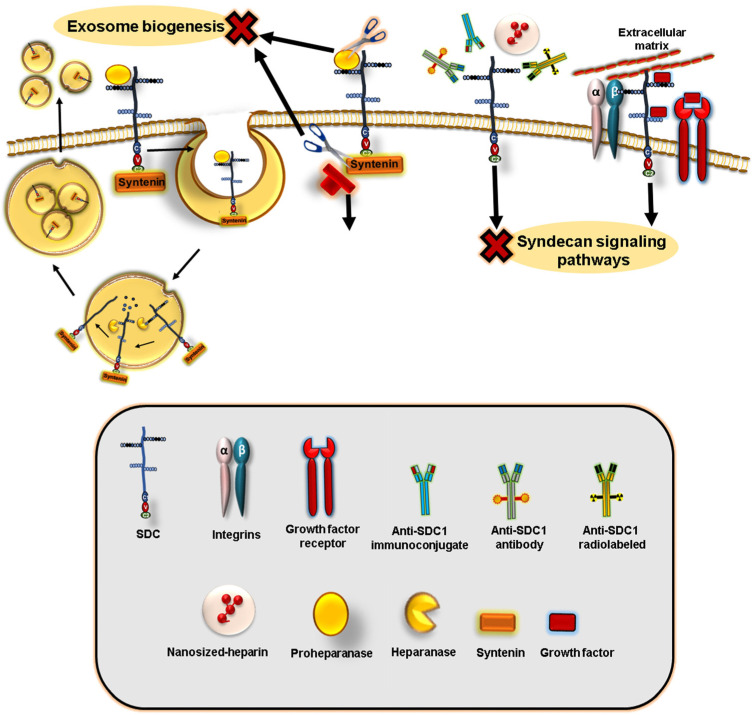
Summary diagram for different therapeutic approaches that can be used for syndecans (SDCs) targeting breast cancer. This includes monoclonal anti-SDC1 antibodies, immunoconjugates linked to cytotoxic drugs, or radiolabeled antibodies (immunoradiotherapy). Further therapeutic perspectives include the application of different types of pharmaceutical agents or inhibitors, such as peptide inhibitors (to impede their interactions with integrins or RTKs), small molecule inhibitors, and HS mimetics (or their nanoformulations, e.g., nanoheparin), or inhibitors for exosomal packing (e.g., SyntOFF inhibitors targeting the PDZ2 domain of syntenin). For further details, please refer to the text.

Given the important and pleiotropic role played by SDCs in cancer development and progression, SDC-centered targeting has emerged as a promising therapeutic regimen [6,68,155,156]. These approaches can include targeting via monoclonal antibodies, peptides, specific pharmacological inhibitors, nanosized heparin, or miRNAs [157,158], as summarized in Figure 4.

### 8.1. Monoclonal Antibodies

It has been demonstrated that treatment with the antibody-drug-conjugate Indatuximab ravtansine (BT062), either alone or combined with docetaxel or paclitaxel to specifically target SDC1 (CD138)-expressing cells, results in marked tumor regression in vitro and in different triple-negative breast cancer patient-derived xenograft models [159]. Another study showed that targeting SDC1/CD138 by employing immuno-PET imaging and radioimmunotherapy (RIT) using the radiolabeled antihuman SDC1 B-B4 significantly induced tumor regression in a xenograft model of triple-negative breast cancer [160]. In a preclinical study, it was demonstrated that preincubating highly metastatic mouse 4T1 breast cancer cells with anti-SDC4 antibodies curtailed the early steps of metastasis in the bone of a mouse model. The prometastatic effect exerted by SDC4 was mediated via its interaction with Autotaxin-β, a member of the nucleotide pyrophosphatases phosphodiesterase family involved in the formation of spontaneous breast tumors and metastasis, suggesting a functional crosstalk between SDC4 and Autotaxin-β [161]. Not only can the targeting of cancer-cell autologous SDC4 be exploited, but it can also be extended to the tumor microenvironment’s endothelial cells. In this regard, it has been reported that the humanized monoclonal antibody trastuzumab used for breast cancer treatment can interact with SDC4, resulting in reduced proliferation, invasion, and angiogenesis of anoikis-resistant endothelial cells [162]. Although it is speculative, a similar mechanism can be exerted in mammary carcinoma cells.

In addition to the protein core of SDCs, the covalently linked GAG chains emerge as important druggable targets as well, given their ability to interact with numerous ligands such as growth factors, cytokines, chemokines, and proteases, influencing cell signaling events implicated in cancer progression [6]. Indeed, the monoclonal antibodies JM-403 and HS4C3 have been developed to recognize and bind HS chains, interfering with ligand tethering capacities in different diseases [163,164]. This may open new avenues for the possibilities of designing antibodies against GAG chains of SDCs on tumor cells. Nevertheless, the GAG chain length and sulfation degree/pattern are context-dependent in various cancers [165]. Thus, GAG chains of SDCs should be characterized in breast cancer to identify and effectively target their different ligand-binding sites.

### 8.2. HS/GAGs Mimetics

Heparin is a highly sulfated, negatively charged, and naturally occurring anticoagulant [166]. It not only prevents venous thromboembolism in cancer patients but also exerts antimetastatic activities by interactions with different receptors and inhibiting tumor progression [167]. However, due to the adverse effects caused by heparin, such as bleeding and thrombocytopenia [168], intense research studies have been performed to develop heparin analogs or HS mimetics with no anticoagulant activities and fewer side effects. In this regard, and based on the impressive data of their anticancer effects in preclinical models, the HS mimetics muparfostat (PI-88), pixatimod (PG545), and necuparanib (M402) entered clinical trial phases I, II, and III alone or in combined therapy to evaluate their safety and beneficial effects on the survival of a patient with different advanced solid cancers and multiple myeloma (reviewed in [165,169]). The mechanism of action of these HS-mimetics is primarily mediated via inhibition of heparanase-mediated angiogenesis and metastasis, competitive binding with growth factors, and/or activation of tumor-suppressing immune cells (e.g., natural killer and dendritic cells) [165,169]. An interesting study showed that in an ex vivo explant model of normal human female mammary tissue, heparanase upregulates the expression and cleavage of SDC1 rather than SDC4, resulting in high mammographic density (a strong and independent risk factor for breast cancer) with a fibrous stroma rich in SDC1. This leads authors to suggest that either single or dual inhibition of heparanase and SDC1 can suppress the risk of breast cancer (progression) in individuals (patients) with high mammographic density [170]. SDC1 can be targeted via the inhibiting activity of heparanase, which is functionally associated with SDC1 [155]. In this regard, roneparstat (SST00001), a chemically modified heparin and an inhibitor for heparanase activity, might have therapeutic potential due to its capacity to disrupt the heparanase/SDC1 axis, as previously described [171,172].

Since nanomedicine has gained great attention in recent years due to the promising results of nano-sized therapeutic agents to combat cancer [173], a prior study expanded the significance of invertebrate-derived heparin and evaluated its anticancer function in its nanoformulation form. Interestingly, nanosized heparin isolated from the sea squirt Styela plicata (ascidian heparin) markedly suppressed breast cancer cell invasion, proliferation, and proteasome activity when compared with nanosized heparin isolated from the porcine intestine (mammalian heparin). Mechanistically, nano-Styela heparin downregulated the expression of ECM-related genes, such as *uPA*, *MT1-MMP*, *SDC1*, and *SDC2*, but not *SDC4* [174].

### 8.3. Pharmacological or Peptide Inhibitors

Since SDC1 has no active site that can be directly inhibited, the therapeutic approaches developed depend on interfering with the interactions of SDC1 with its partner proteins. Indeed, a novel peptide inhibitor, synstatin, corresponding to the site’s core protein of SDC1, was developed to hinder the interaction between SDC1 and both integrin α_v_β_3_ and α_v_β_5_. Systemic administration of synstatin retarded tumor growth in a xenografted mice model with MDA-MB-231 cells and corneal angiogenesis in vivo, reinforcing the role of SDC1 in regulating α_v_β_3-_ and α_v_β_5_-induced tumorigenesis and angiogenesis [37]. Preclinical and clinical studies have addressed the anticancer activity of third-generation bisphosphonates (BP) and zoledronate (zoledronic acid, Zometa^®^). Mechanistically, BPzoledronic acid exhibits an anti-breast cancer effect, namely the inhibition of cell proliferation, adhesion, migration, and invasion via the suppression of the expression of SDC1 and 2, whereas SDC4 was upregulated [175]. Further, the broad-spectrum MMP inhibitor GM6001 curtails breast carcinoma cell growth by blocking stromal SDC1 shedding [176].

Interference with SDC1 expression can influence multiple and interconnected signaling pathways relevant to breast cancer progression. We have previously shown that SDC1 expression affects cancer stem cell-related pathway components, including IL-6/STAT3, Notch, and EGFR signaling pathways, in SUM-149 triple-negative inflammatory breast cancer cells, an aggressive and deadly form of breast cancer [69]. Therefore, SDC1 targeting alone or in combination with these pathways’ inhibitors emerges as a promising therapeutic regimen for this devastating disease. On the other hand, SDC1 is affected by Notch signaling. A study reported that the SDC1 transmembrane C-terminal fragment cleaved by ADAM17 undergoes intra-membrane proteolysis in a gamma-secretase-dependent mechanism [177]. Thus, the use of gamma-secretase inhibitors may serve simultaneously to target both Notch and SDC1-regulated pathways [69,178].

It has been shown that SDC2 is another attractive target in breast cancer. Previous data indicate that the inhibition of epithelial or stromal SDC2 with SDC2 peptides retards breast tumor growth and metastasis, as well as retraining tumor evasion (via downregulation of TGFβ-regulated programmed death ligand 1 and *CXCR4*) in a xenograft mouse model [41,58].

Constitutive and induced expression of SDC2 and 4 are affected by the coordinated crosstalk between the RTK EGFR and IGFR with ERs in vitro in MCF-7 and ERβ-positive MDA-MB-231 breast cancer cells [91]. Therefore, multitarget pharmacological inhibitors can be developed to treat breast cancer that exhibits resistance to endocrine therapy. Imatinib, an inhibitor of several tyrosine kinases, including BCR-ABL, c-KIT, and PDGF-Rs, significantly mitigated PDGF-induced breast cancer cell proliferation, invasiveness, and migratory capacity, associated with downregulation of SDC2 and 4 expressions [158,179]. A recent study showed that the cell cycle of triple-negative breast cancer cells is regulated by a complex formed by the docking of EGFR with the extracellular domain of SDC4 (engaged with α3β1 and α6β4 integrins), followed by the incorporation of SDC2, RON tyrosine kinase, and ABL1. Independent of EGFR kinase activity, RON kinase activates ABL1 to arrest the cell cycle via the inhibition of p-38 MAPK signaling. Therefore, from a therapeutic perspective, a peptide mimetic (SSTN_EGFR_) of the EGFR docking site in SDC4 can be used to disrupt the formation of this complex with subsequent cell cycle arrest [180].

### 8.4. Exosome Targeting-Based Therapies

Other approaches to exosome-targeted therapy have emerged. A very interesting recent study indicated that out of 139 in silico screening compounds, a novel non-toxic SyntOFF inhibitor targeting the PDZ2 domain of syntenin was found to impede exosomal cargo loading with syntenin, ALIX, and SDC4 accompanied by inhibitory effects on cell proliferation, mammosphere formation, and migration of MCF-7 cells [181]. Similarly, small molecule inhibitors were developed to target the interaction between the syntenin PDZ2 domain and SDC2 in MCF-7 cells, affecting the subsequent release of exosomes [182]. In addition, heparanase is involved in regulating SDC1 loading in exosomes, evident by the high content of SDC1 in exosomes secreted by heparanase-high expressing cells vs. heparanase-low expressing cells, with subsequent impacts on endothelial cells and cancer progression [183]. In this regard, exosomes did not only mediate the cancer cell-autonomous effect but extended to the microenvironmental endothelial cells. Therefore, the interference with the biogenesis and action of exosomes (possibly via dual heparanase and SDC1 targeting, as we have indicated above) could have an excellent impact at multiple levels of cancer hallmarks. Intriguingly, genetic-based evidence indicates that, in general, the cell surface HSPGs are key receptors for cancer cell-derived exosomes’ internalization and functional activity [184], suggesting that interference with the expression of SDCs could represent a therapeutic strategy to impede the delivery of exosome cargo and subsequent organotropic metastasis, as we described earlier. Given the relevant role played by SDC1 in lung tumorigenesis via regulation of the exosomal miRNA pattern [185], we propose a similar mechanism that may exist in breast cancer. However, the role of SDCs in this context is yet poorly investigated, and further future research in this area is needed.

### 8.5. Other SDCs Targeting Therapeutic Modalities

Other therapeutic strategies can include different biomolecule derivatives or mimetics. For example, different molecular weights of hyaluronan (HA), a major ECM constituent, appear to have therapeutic potential against breast cancer. Indeed, treatment of MDA-MB-231 (highly metastatic) and MCF 7 (low invasiveness) cells with different molecular sizes of HA fragments to compete with endogenous HA resulted in elevated mRNA and protein levels of SDC4, which is responsible for enhanced cell adhesion and inhibition of cell invasion into collagen and aggressiveness [186].

miRNA-centered therapeutic approaches have undergone clinical trials I/II showing their potency against diseases, including cancers [128]. As we mentioned above, SDC expression is directly or indirectly regulated by a wide range of miRNAs, and subsequently, SDC-targeted miRNA mimetics hold great promise for breast cancer therapy. Nevertheless, it should be noted that multiple targets regulated by miRNAs are dependent on cell type, and it is difficult to ascribe miRNA effects to one target.

## 9. Conclusions

A growing body of research employing preclinical and clinical settings unveiled the intricate roles of SDCs in breast cancer progression, namely: (i) their dysregulated expression in breast cancer cells/tissues and tumor-associated cells (e.g., fibroblasts); (ii) their (co)receptor functions for RTKs and integrins and interactions with ECM components; (iii) their interplay with components of numerous signaling pathways (such as ERα, ERβ, AR, PPAR-gamma, and cancer stem cell-related molecules) relevant to breast cancer progression-associated processes (e.g., proliferation, adhesion, migration, invasion, metastasis, and EMT); (iv) their regulatory role in exosome biogenesis and functioning; and (v) their function as miRNA targets or upstream regulators of miRNA expression. Together, these mark SDCs as signaling mediators, prognostic markers, and therapeutic targets for breast cancer. Given the evaluation of cell surface PGs in clinical trials for different tumor settings, SDC family members appear to be important candidates for therapeutic targeting using monoclonal antibodies (given their plasma membrane expression), peptide inhibitors (to impede their interactions with integrins or RTKs), small molecule inhibitors, biomolecule mimetics (e.g., miRNAs and HMWHA), and HS mimetics (or their nanoformulations) or inhibitors for their exosomal packing. SDC targeting approaches are encouraging for translation into clinical practice for breast cancer therapies. However, some aspects should be taken into consideration for SDC targeting. For example, circulating shed SDCs which can neutralize the therapeutic efficacy of systemic SDC-targeting antibodies. This challenge can be overcome by developing antibodies that only recognize intact SDCs rather than shed SDCs [187]. Another problem is the expression of SDCs on normal tissues, which may restrain their prospective translation as therapeutic targets. Therefore, it is important to design antibodies to target SDCs with enhanced “on-target” effects and reduced “off-tumor” toxicity [188]. This can be achieved by pH-responsive antibodies (being able to bind to their target antigens, exploiting the acidic pH of the tumor microenvironment) [187,189] or by enhancing tumor-targeting selectively using antibodies with high affinity for two antigen targets and with low affinity for single targets, the so-called bispecific antibody [187,190]. For example, SDC1 is overexpressed in metastatic HER2-overexpressing breast cancer [191]. Trastuzumab (Herceptin^®^) is an FDA-approved monoclonal antibody used to treat HER2-overexpressing breast cancer patients [192]. As such, the modification of already available trastuzumab or the development of a new bispecific antibody to target both SDC1 and HER2 may open a therapeutic window and improve the patient outcome for metastatic breast cancer. The expression patterns and localization (membranous, cytoplasmic, or stromal) of SDCs in breast cancer subtypes are varied. Therefore, clinical trials are needed to stratify breast cancer patients who would benefit from SDC targeting either alone or in combination therapies.
